# Deposition of an ultra-thin polyaniline coating on a TiO_2_ surface by vapor phase polymerization for electrochemical glucose sensing and photocatalytic degradation†

**DOI:** 10.1039/d0ra01571g

**Published:** 2020-05-05

**Authors:** Sibani Majumdar, Debajyoti Mahanta

**Affiliations:** Department of Chemistry, Gauhati University Assam India 781014 debam@gauhati.ac.in debam2@gmail.com +91-0361-2700311 +91-6000682511

## Abstract

Here, we have synthesized an ultra-thin coating of polyaniline on a TiO_2_ nanoparticle surface (PANI–TiO_2_) using a simple vapor phase polymerization method. By this method, an ultra-thin layer of PANI is obtained selectively on the TiO_2_ surface. This ultra-thin coating exhibits the properties of both the parent materials due to the composite surface causing an effective synergistic effect. SEM, TEM, and EDX studies and elemental mapping confirmed the formation of ultra-thin films on the TiO_2_ surface. TGA, UV/Vis and XRD studies were also done for further characterization. The composite has been used as a biosensor for glucose detection by immobilization of the enzyme glucose oxidase (GOx). Cyclic voltammetry, electrochemical impedance spectroscopy and amperometry studies were performed for glucose sensing. The linear range was observed from 20 to 140 μM glucose concentration from the amperometric analysis. The LOD of the biosensor was found to be 5.33 μM. The composite has also been used for photocatalytic degradation of the cationic dye Rhodamine B (RB). The order of degradation efficiency of RB is found to be PANI < TiO_2_ < PANI–TiO_2_. The synergetic effect of PANI and TiO_2_ is the reason for the enhanced degradation efficiency of the composite PANI–TiO_2_.

## Abstract 2

Here, we have synthesized an ultra-thin coating of polyaniline on a TiO_2_ nanoparticle surface (PANI–TiO_2_) using a simple vapor phase polymerization method.

## Graphical abstract

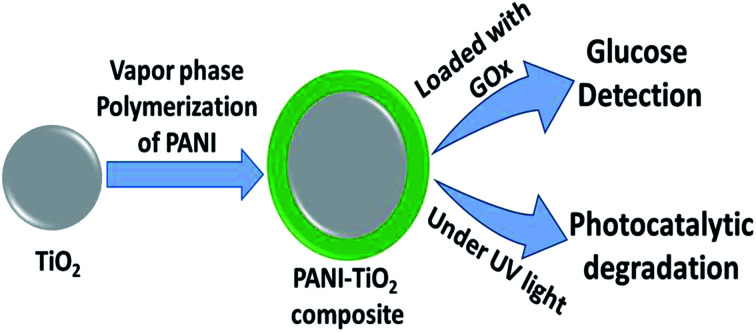

## Introduction

Titanium dioxide (TiO_2_) has a wide range of applications such as in photovoltaic cells, drug delivery systems, and photocatalytic degradation of various pollutants and biosensors. Due to its promising structural, electrical and optical properties, it has received a lot of attention in recent years. Various advanced and hybrid materials of TiO_2_ have been designed to improve the properties of the parent material for superior applications. One of the uses of TiO_2_ is in biosensors for detection of glucose.^1^ There has been a rapid growth in the development of precise and convenient glucose monitoring systems in recent years. More than 85% of the biosensor market is occupied by glucose biosensors which indicate the demand of low-cost glucose sensors with high sensitivity and selectivity to deal with increasing blood sugar problem. Most of the biosensors available in market are electrochemical biosensor immobilized with a specific enzyme. Glucose oxidase (GOx) is the most popular and commercially used enzyme in enzyme-based glucose sensors. According to WHO, in 2012 around 1.5 million death is caused by diabetes.^2^ Diabetes also leads to kidney failures, vision loss and cardiovascular disease. In enzymatic biosensor, the enzyme glucose oxidase converts glucose into gluconolactone and generates hydrogen peroxide as a co-product.^3^ The first generation of glucose sensor involves measurement of the formed hydrogen peroxide. In the second generation sensors, a mediator is used for the transfer of electrons between the electrode and enzyme active site, while in third generation of glucose sensor, direct electron transfer takes place. In the third generation of glucose biosensors, the enzyme has been immobilized on different materials to facilitate the direct electron transfer.^2^ Semiconductors such as zinc oxide, zirconia and titanium dioxide are some of the choices of researchers as electrode materials for electrochemical biosensors.^3–5^ Various studies have been reported on TiO_2_ as glucose sensing material because of its high specific surface area, porous structure and a good biocompatibility.^6,7^ But poor electrical conductivity hinders its application in more precise and sensitive biosensor. Therefore, hybrid materials of TiO_2_ with other organic and inorganic materials have drawn the attention of the researchers for better results.^8–10^ Conducting polymers specially polyaniline (PANI) has been established as efficient electrode material in enzyme-based sensors due to its high electrical conductivity and high stability.^11,12^ But some limitations such as weak mechanical properties and poor biocompatibility affect the performance of the PANI based biosensors.^13^ However, composites of PANI with some of the materials can enhance its applicability as biosensor.^14,15^ Composites of two materials PANI and TiO_2_ have been considered as a promising class of materials to overcome the limitations of parent components.^16,17^

Oxidation of pollutants is one of the wastewater treatment techniques that have been used for degradation of several organic contaminants. Photocatalytic degradation is also an oxidation technique, in which free radicals are generated by interaction of catalyst with photons of proper energy. TiO_2_ has been used as an efficient photocatalyst from many years for degradation of a wide range of pollutants under UV radiation.^18^ The photocatalytic activity of semiconductor like TiO_2_ was discovered by Frank and Bard where they have studied the degradation of cyanide.^19^ Later, Ollis *et al.* studied the potential application of photocatalysts for degradation of various organic compounds.^20^ When electromagnetic radiation of specific energy illuminated on the photocatalyst surface, excitation of valence band electrons to the conduction band takes place. This leads to the formation of positive hole and electrons in the valence band and conduction band respectively. The positive hole can directly oxidize the pollutants, or it may indirectly oxidize water to produce hydroxyl radicals that degrade the pollutants.^21^ The electron at the conduction band of the photocatalyst reduces the adsorbed oxygen. To keep the activity of the photocatalyst, simultaneous oxidation of the pollutants and reduction of the oxygen is necessary to avoid the recombination of the positive holes and electrons.^21,22^ To enhance the photocatalytic activity of the TiO_2_ based catalyst, researchers are working on modification of the catalysts using various strategies such as metallization, doping and sensitization *etc.*^23^ Conducting polymers acts as photosensitizer to improve the photocatalytic activity of TiO_2_ based catalysts.^24–26^ The various promising properties of PANI and its suitable band gap to sensitize TiO_2,_ attracted the researchers to develop PANI–TiO_2_ composites for photocatalytic degradation of organic pollutants.

Here, we have prepared PANI–TiO_2_ composite by deposition of ultra-thin coating of PANI on TiO_2_ surface by vapor phase polymerization. PANI has been synthesized selectively on the surface of TiO_2_ particles by this easy and simple technique. The synthesized material has shown good photocatalytic activity towards the degradation of dye and glucose sensing. Rhodamine B (RB) a cationic dye has been effectively degraded by this composite. The composite shows enhanced degradation in comparison to bare TiO_2_. The synergetic effect of PANI and TiO_2_ is the reason for enhanced activity of this composite for glucose sensing as well as photocatalytic degradation of organic pollutant.

## Results and discussions

### Characterizations

**Fig. 1:**
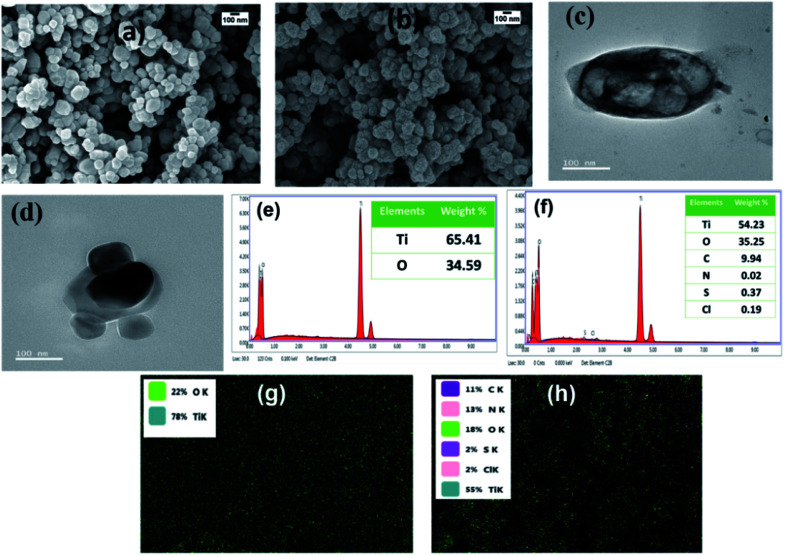
SEM images of (a) TiO_2_ and (b) PANI–TiO_2_, TEM images of PANI–TiO_2_ (c and d), EDX pattern of (e) TiO_2_ and (f) PANI–TiO_2_ and electron-dot mapping of (g) TiO_2_ and (h) PANI–TiO_2_.

Fig. 1(a) and (b) show the SEM images of TiO_2_ and PANI–TiO_2_, respectively. It is observed from the micrographs that PANI is coated on the surface of the TiO_2_ nanoparticles uniformly. It is observed from the SEM that the average size of both TiO_2_ and PANI–TiO_2_ particles are 80–100 nm. As there is no noticeable change in the shape and size of TiO_2_ particles after deposition of PANI, it confirms the advantage of vapor phase polymerization technique for ultra-thin and uniform coating of PANI on TiO_2_ surface. The enlarged SEM images of selected portions were compared and uniform layer of granular shaped PANI is observed in the case of PANI–TiO_2_ which is absent in TiO_2_ (Fig. S1†). The ultra-thin layer coating of PANI on the TiO_2_ particles are further confirmed from the TEM analysis of the composite given in Fig. 1(c) and (d). Fig. 1(e) and (f) shows the EDX pattern and the corresponding element weight% of bare TiO_2_ and the composite. From the EDX analysis it is observed that TiO_2_ consist 65.41% of Ti and 34.59% of O. While in the EDX of PANI–TiO_2_ composite has Ti (54.23%), O (35.25%), C (9.94%), N (0.02%), S (0.37%) and Cl (0.19%). It confirms the presence of PANI on the PANI–TiO_2_ composite. The S content is due to APS used as oxidant for synthesis of PANI. Generally, the weight% of N is high in PANI, but here in the PANI–TiO_2_ composite the weight% found for N is quite low. The peak for N is observed near 0.4 keV and the Ti peak is also appeared in the same range. So, it may overlap the peak corresponding to N.

Therefore, we have also performed the electron-dot mapping analysis of TiO_2_ and the composite for confirmation of PANI coating. It is observed from elemental mapping shown in Fig. 1(g) and (h) that TiO_2_ has only Ti and O whereas PANI–TiO_2_ possesses all the elements of PANI (C, N, Cl and S) along with Ti and O. Thus, uniform and thin coating of PANI on the surface of TiO_2_ is confirmed from the SEM, TEM, EDX and elemental mapping study.

**Fig. 2:**
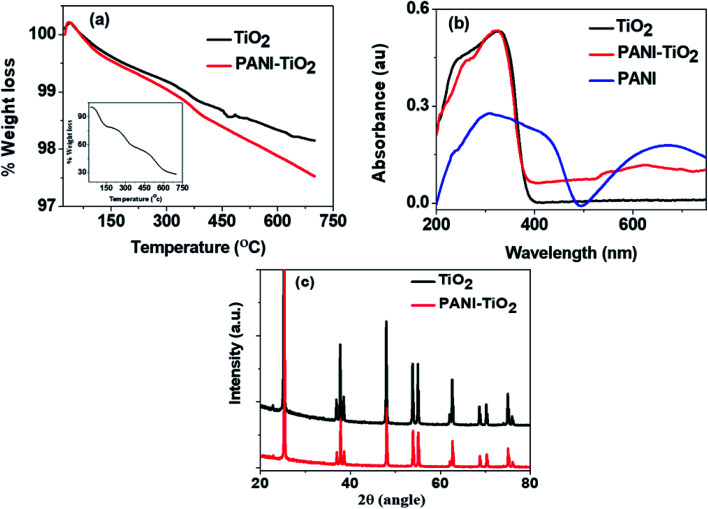
(a) Thermogravimetric analysis (inset shows the TG of PANI) (b) diffuse-reflectance UV-Visible spectra and (c) XRD pattern of TiO_2_ and PANI–TiO_2_ composite.

Fig. 2(a) shows the thermograms of TiO_2_ and PANI–TiO_2_ in the temperature range of 25–700 °C and the inset shows the TG of bulk PANI synthesized by conventional chemical oxidative polymerization (SI-1). It is observed that TiO_2_ possesses a high thermal stability and only 2% weight loss is there up to 700 °C while the degradation of PANI is more and about 72% weight loss up to 700 °C [inset Fig. 2(a)]. Similar results were also found in some other studies.^27,28^ The degradation pattern of PANI–TiO_2_ is also similar to that of TiO_2_ but the degradation is slightly more than that of TiO_2_ which started around 200 °C. This increase in degradation of the composite is due to the degradation of the PANI. It is interesting to note that only 1% more degradation is observed in PANI–TiO_2_ which indicates the presence of very low amount PANI in PANI–TiO_2_ composites.

The UV-Visible diffuse reflectance spectra of TiO_2_, PANI–TiO_2_ and PANI (synthesized by chemical polymerization, SI-1) are shown in Fig. 2(b). The characteristics bands of PANI are observed near 300 and 650 nm. TiO_2_ absorbs light only below 400 nm with the absorption sharp edge near 385 nm. The PANI–TiO_2_ composite also shows a similar band below 400 nm with absorption sharp edge near 380 nm. The peak observed at 650 nm in PANI is almost disappeared in the spectrum of PANI–TiO_2_ composite, rather a broad absorption band appeared near 600 nm which is absent in the absorption spectrum of bare TiO_2_. This broad band appears due to the charge carrier delocalization of PANI. Thus, the composite is expected to generate more electron–hole pairs than that of both PANI and TiO_2_, which will increase its photocatalytic activity.^29,30^

The highly crystalline structure of TiO_2_ is observed in the XRD pattern as shown in Fig. 2(c). The Bragg diffraction peaks indexed as (101), (112), (200), (105), (211), (204), (116), (220) and (215) indicates the anatase phase of TiO_2_ having tetragonal arrangement.^31^ The diffraction pattern of the synthesized PANI–TiO_2_ composite is also similar to that of TiO_2_ and does not show any characteristic pattern of PANI. The highly crystalline nature of TiO_2_ may overshadow the characteristic low intensity peaks of PANI. Moreover, the amount of PANI present in the composite is extremely small (around 1%) as compared to TiO_2._ However, it is noticed that the surface modification by PANI does not affect the structure of TiO_2_.

### Electrochemical analysis for glucose detection

**Fig. 3:**
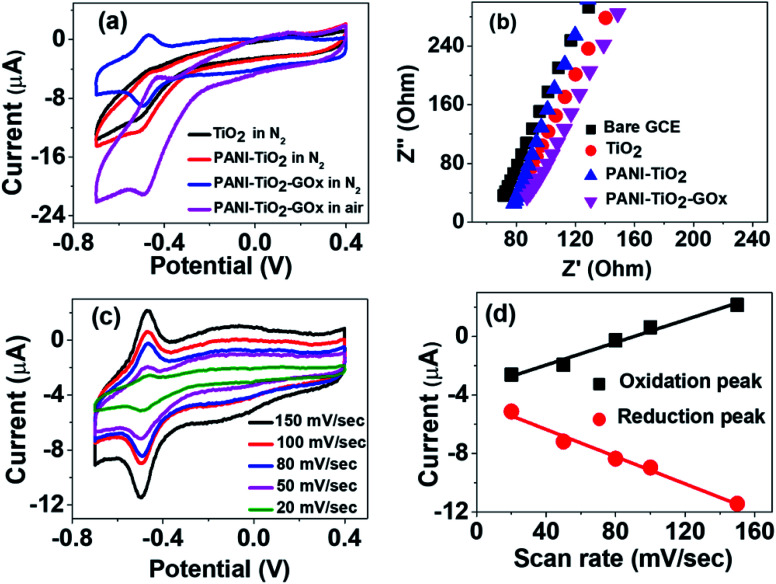
(a) CVs of TiO_2_, PANI–TiO_2_ and PANI–TiO_2_–GOx in air and N_2_ saturated 0.1 M PBS (b) EIS in N_2_ saturated 0.1 M PBS (c) CVs of PANI–TiO_2_–GOx at different scan rate (d) plot of oxidation and reduction current *vs.* scan rate.

Fig. 3(a) shows the CVs of TiO_2_, PANI–TiO_2_ and PANI–TiO_2_–GOx at a scan rate of 100 mV s^−1^ in 0.1 M N_2_ saturated PBS. It is observed that the redox peak current of the PANI–TiO_2_ coated electrode is more than that of the TiO_2_ electrode. It is obvious because PANI enhances the conductivity in PANI–TiO_2_ composite in comparison to bare TiO_2_ electrode. Again, after loading the enzyme on PANI–TiO_2_ composite, the redox peaks become more distinct but the current decreases as GOx is a biomacromolecule introducing more electrical resistance.^17^ This result is also supported by electrochemical impedance studies as shown in Fig. 3(b). Due to the higher conductivity of the bare GCE, the charge transfer resistance (R_ST_) of TiO_2_ coated electrode is more than that of the bare GCE. Then, the charge transfer resistance decreases on the PANI–TiO_2_ coated electrode due to the incorporation of conducting polymer PANI. Thus, PANI helps in enhancing the charge transfer in PANI–TiO_2_ composite electrode. The charge transfer resistance again increases in the PANI–TiO_2_–GOx coated electrode due to GOx which is an electrical insulator.

Due to the introduction of GOx as described above, it is observed from Fig. 3(a) that, two almost symmetrical redox peaks appeared at around −0.49 V (reduction peak) and −0.46 V (oxidation peak) on the CV of PANI–TiO_2_–GOx electrode. This indicates the direct electron transfer of the enzyme with the electrode surface. Thus, the GOx is adsorbed on the composite surface and involves in reversible electrochemical process as shown in eqn (1).^32^1
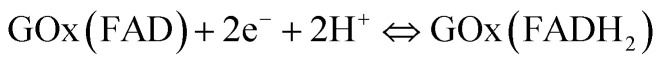


We have also compared the electrochemical activity of the PANI–TiO_2_–GOx composite with air saturated 0.1 M PBS. The peaks appeared are not symmetrical when the PBS is not saturated with N_2_. In presence of oxygen, the chemical reaction given in eqn (2) takes place at the electrode surface. Thus, the dissolved O_2_ again oxidizes the reduced enzyme and increases the reduction peak current.^32,33^

2 GOx(FADH_2_) + O_2_ → GOx(FAD) + H_2_O_2_

To study the effect of scan rate on the electrochemical behavior of the composite, we have carried out the CV experiments in N_2_ saturated PBS by varying the scan rate from 20 to 150 mV s^−1^ as shown in Fig. 3(c). It is observed that the redox peak current value changes linearly with the scan rates. The linear relationship of the scan rate with redox peak current value is shown in Fig. 3(d). This linear relationship indicates that the whole process is limited by the adsorption of the composite on the electrode surface and not because of diffusion.^17^

**Fig. 4:**
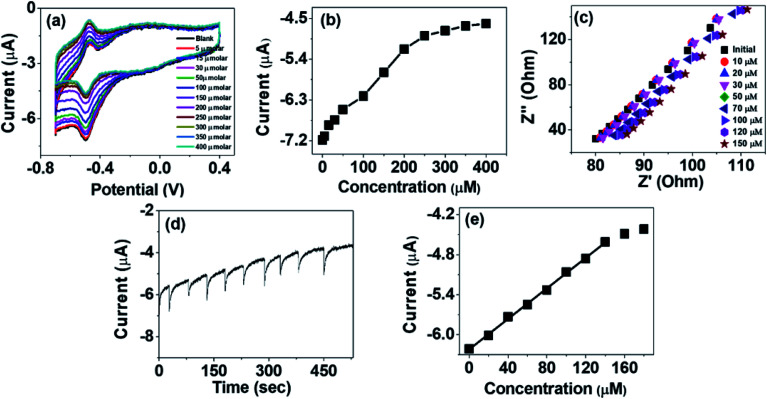
(a) CVs of PANI–TiO_2_–GOx in N_2_ saturated 0.1 M PBS at different glucose concentration (b) plot of reduction current *vs.* glucose concentration (c) EIS of PANI–TiO_2_–GOx electrode at different glucose concentrations (d) amperometric response of PANI–TiO_2_–GOx electrode for different glucose concentration at −0.5 V (e) calibration plot of the sensor electrode from the amperometry measurement.

The activity of PANI–TiO_2_–GOx electrode for glucose sensing was studied by investigating the CVs of the electrode in N_2_ saturated PBS at a constant scan rate of 50 mV s^−1^ at different concentrations of glucose solution (5 to 400 μM). It has been observed that addition of glucose greatly affects the reduction peak current which is observed at around −0.49 V. On addition of glucose the reduction peak current decreases as shown in Fig. 4(a). It has been observed that the decrease of the peak current is linear up to a concentration of 200 μM. Beyond that concentration, the change is not linear as the active centers may get started to saturate as observed in Fig. 4(b). The decrease of the reduction peak current on addition of glucose can be explained by eqn (3)^13,14,32^

3 GOD(FAD) + Glucose → GOD(FADH_2_) + Gluconolactone

With increase in the glucose concentration, the conversion of GOD(FAD) to GOD(FADH_2_) increases resulting in decreasing concentration of GOD(FAD) and consequently the reduction peak current decreases. The decrease of the current can also be explained by the impedance spectra of PANI–TiO_2_–GOx (Fig. 4(c)) as the charge transfer resistance (*R*_ST_) of the composite electrode increases on addition of glucose.

The amperometric measurement was done at a constant potential of −0.5 V by adding successive 20 μM increase in glucose concentration as shown in Fig. 4(d) and the calibration plot obtained is given in Fig. 4(e). The linear range was observed from 20 to 140 μM with a linear equation of *I* = 0.01142*x* − 6.22. The sensitivity of the biosensor was calculated to be 163.14 μA mM^−1^ cm^−2^ from the slope of the calibration plot and area of the electrode surface.^13^ The limit of detection (LOD) has been calculated using the eqn (4)^14^4
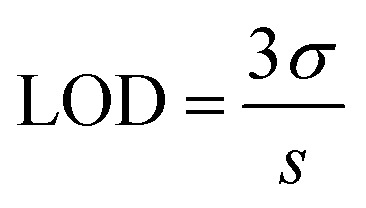
where *s* is the slope and *σ* represents the standard deviation of the response. The LOD of the biosensor was found to be 5.33 μM. The LOD, sensitivity and preparation methods of some of the TiO_2_ based hybrid composites are given in Table 1. From the table it is observed the developed composite has low LOD value with a decent sensitivity.

**Table 1:** LOD values, sensitivity and preparation methods of some of the TiO_2_ based hybrid composites

Material	Preparation method	LOD	Sensitivity	Ref.
Carbon nanotubes modified titania nanotube arrays, fabricated with platinum nanoparticles	Vapor-growing in the inner of titania NTs	5.7 μM	0.24 μA mM^−1^ cm^−2^	9
Hierarchical one-dimensional TiO_2_	Solvothermal method using MWCNTs as template	1.29 μM	9.90 μA mM^−1^ cm^−2^	10
Polyaniline–TiO_2_ nanotube	Hydrothermal and oxidative polymerization	0.5 μM	177.16 μA mM^−1^ cm^−2^	13
Polyaniline/active carbon and nanometer-sized TiO_2_ composite	Oxidation and sol–gel method	18 μM	6.31 μA mM^−1^ cm^−2^	17
GOD–PPy/TiO_2_ nanotube	Anodic oxidation and normal pulse voltammetry process	1.5 μM	187.28 μA mM^−1^ cm^−2^	34
GOD–nanoporous TiO_2_ film–carbon nanotube composite	Sol–gel method followed by vapor chemical deposition of CNT	30 μM	8.08 μA mM^−1^ cm^−2^	35
PANI–TiO_2_ composite	Vapor phase polymerization	5.33 μM	163.14 μA mM^−1^ cm^−2^	Present study

### Photodegradation studies

**Fig. 5:**
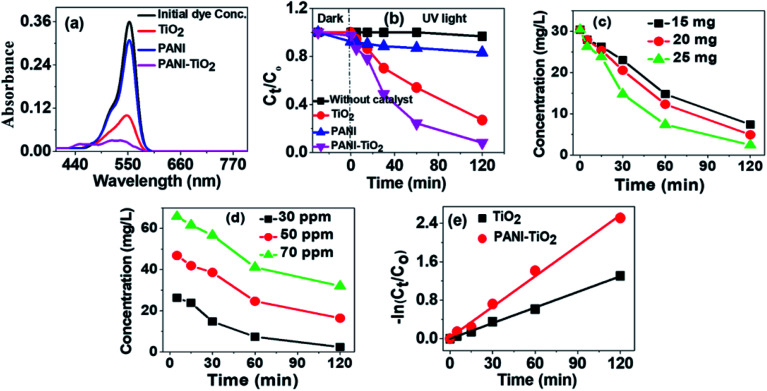
(a) Absorption spectra of RB after 2 h of degradation using TiO_2_, PANI and PANI–TiO_2_ (b) concentration profile of RB at different time interval in dark and under UV light without catalyst, with TiO_2_, PANI and PANI–TiO_2_ (c) concentration profile of RB with PANI–TiO_2_ with different catalyst amount (d) variation of RB concentration with different initial dye concentrations (e) L–H kinetic model plot for TiO_2_ and PANI–TiO_2_.

To find out the efficiency of PANI–TiO_2_ as photocatalyst and to compare its efficiency with polyaniline emeraldine salt (PANI) and TiO_2_ for degradation of RB, we have performed the degradation experiment. Fig. 5(a) shows the absorption spectra of RB before and after 2 h of degradation using TiO_2_, PANI and the PANI–TiO_2_ composite. It is observed that PANI degrades a very small amount of RB, as it is reported that due to electrostatic interaction PANI is a good adsorbent for anionic molecules only. As RB is a cationic dye, the probability of the reduction of the intensity of dye peak in UV/Vis spectra due to adsorption is negligible. TiO_2_ shows degradation of RB and on using PANI–TiO_2_ the degradation increases significantly. Thus, a small amount of PANI can effectively increase the photocatalytic ability of TiO_2_. The concentrations of RB at different time interval in presence of various photocatalysts were determined. In each experiment, the photocatalyst was mixed to the solution and was stirred in dark for 30 min. Then the solution was irradiated with UV light and collected samples at regular time interval. After diluting the solution, the catalyst was separated by centrifugation. The concentrations of the solutions were determined by UV/Vis spectroscopy using Beer's Lambert law. Fig. 5(b) shows the concentration profile of RB at different time intervals. The dye solutions were stirred in dark after adding the catalysts before exposing it to the light to obtain a state of equilibrium adsorption and also to see the effect of adsorption. The concentration was measured after 30 min adsorption in dark and a very small extent of adsorption has been found for TiO_2_ and PANI–TiO_2_. Thus, the decrease in concentration of RB by PANI–TiO_2_ is primarily due to photocatalytic degradation and not for adsorption. The degradation of dye solution without using any catalyst was also checked as control experiment.

The degradation efficiency is determined by using eqn (5)^36^5
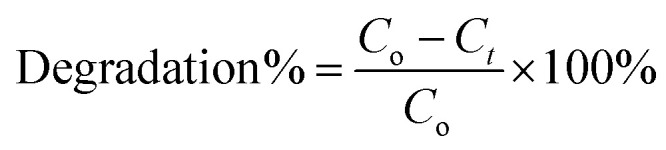
where *C*_o_ and *C*_*t*_ are the initial concentration and the concentration of the dye solution at time *t*, respectively. It is found that the degradation efficiency of the dye without using any catalyst is about 3%. The degradation efficiency of RB with PANI, TiO_2_ and PANI–TiO_2_ are 16.7%, 72.9% and 91.8% respectively. Thus, PANI has increased the efficiency of TiO_2_ as a photocatalyst by synergistic effect. We have also studied the effect of catalyst dose and initial concentration of the dye on degradation. Fig. 5 (c) and (d) shows the effect of catalyst dose and initial concentration on RB degradation by PANI–TiO_2_. It is observed that with increase in both the dose and initial concentration the degradation increases. With increase in catalyst dose the number of active sites increases and with increase in dye concentration the number of molecules accessing the fixed number of active sites increases. Both are responsible for increase in photocatalytic degradation.

To quantify the degradation, kinetic study is important. We have fitted the experimental data of 30 ppm RB degradation by TiO_2_ and PANI–TiO_2_ in the Langmuir–Hinshelwood (L–H) kinetics model as shown in Fig. 5(e). The linear form of the L–H kinetic model is given in eqn (6).^37^6
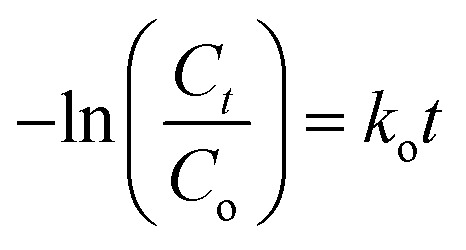
where, *C*_o_ is the initial dye concentration, *C*_*t*_ is the concentration at time *t* and *k*_o_ is the rate constant in min^−1^. Both the kinetic plots have *R*^2^ value of 0.99 and the rate constants obtained from the kinetic plots are 0.01084 and 0.02122 min^−1^ for TiO_2_ and PANI–TiO_2_, respectively. The rate constant of PANI–TiO_2_ is 1.95 times higher than that of TiO_2_.

**Fig. 6:**
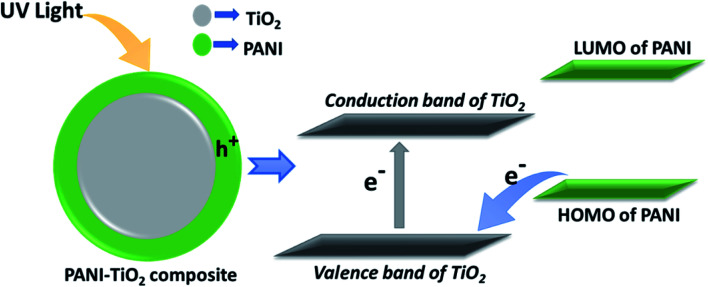
Schematic representation of the possible photodegradation mechanism.

There may be several approaches to enhance the photocatalytic activity of semiconductors like TiO_2_. Some of such approaches are reduction of electron hole recombination, enhancement of efficiency of charge separation, reduction of particle size *etc.* In our study the, enhancement of photocatalytic activity of PANI–TiO_2_ under UV radiation is supposed to be due to the enhanced charge separation because of the synergistic effect of the composites.^38^ The HOMO level energy of PANI is less than the conduction band but more than the valence band energy levels of TiO_2_. On irradiation of UV light, electron is transferred from the valence band to the conduction band of TiO_2_ creating a hole in the conduction band. Electron can directly transfer from the higher energy HOMO of PANI to neutralize the conduction band hole of TiO_2_. This process creates a new hole in HOMO of PANI. As PANI is a good hole transporting material, the hole can easily transport to the catalyst surface to oxidize directly the adsorbed pollutant molecule. The schematic diagram of the possible mechanism is shown in Fig. 6.

## Conclusion

A simple solvent free method for ultra-thin deposition of PANI on TiO_2_ surface by vapour phase polymerization has been demonstrated. It was observed that only around 1% PANI on TiO_2_ surface has significantly enhanced the property of the composites for use in glucose sensor electrodes and photocatalysis. The glucose sensor constructed by the PANI–TiO_2_ composite shows a linear range of glucose sensing from 20 to 140 μM. The LOD value of the PANI–TiO_2_ composite has been calculated to be 5.33 μM. Furthermore, the composite shows enhanced photocatalytic activity under UV light for degradation of cationic dye rhodamine B. The efficiency for the dye degradation with PANI, TiO_2_ and PANI–TiO_2_ are 16.7%, 72.9% and 91.8% respectively for a definite volume and constant initial dye concentration. This result shows the superior activity of the composite in comparison to both the parent materials. This is due to the synergistic effect of the composite materials. Thus, this article reveals the possibility of preparing polymer nanocomposites of superior surface properties by vapour phase polymerization.

## Experimental section

### Chemicals

Titanium dioxide (TiO_2_), ammonium persulphate (APS), hydrochloric acid (HCl), phosphate buffer solution of pH 7.4 (PBS), glucose, glucose oxidase (GOx) and rhodamine B were bought from Merck, India and used as received. Aniline was also purchased from Merck, India and distilled before use. In all the experiments double distilled water was used.

### Preparation of PANI–TiO_2_ composite

We have used the vapor phase polymerization technique for the preparation of the PANI–TiO_2_ nanocomposites. At first, an APS solution was prepared by adding 2.45 g of APS in 20 mL 1 M HCl solution. Then, 100 mg TiO_2_ was added to the prepared APS solution and stirred for 1 h for adsorption of the oxidizing agent on the TiO_2_ surface. After that the APS treated TiO_2_ was dried in room temperature for 1 h, covered with a dry filter paper and placed in a chamber to get exposed by the aniline vapor. A low-pressure environment was maintained inside the chamber throughout the experiment by using vacuum pump. The polymerization takes place on the surface of the TiO_2_ and exposure of aniline vapor was continued for 24 h for complete polymerization. The resultant material was washed with distilled water and kept in desiccator for drying at room temperature.

### Characterization techniques

The powder X-ray diffraction (PXRD) was carried out using a Rigaku Ultima IV powder diffractometer using Cu-Kα X-radiation (*λ* = 1.5406 Å). The scanning electron microscopy (SEM), energy dispersive X-ray (EDX) and the elemental mapping of the materials was carried out with a Zeiss Ultra 55 model. A Mettler Toledo thermogravimetric analyser has been used to carry out the thermogravimetric analysis (TGA). UV-4100 Hitachi spectrophotometer has been used to study the diffuse-reflectance UV/Visible spectra. The transmission electron microscopy (TEM) of the composite was carried out using a field emission transmission electron microscope (FETEM) (JEOL, model: 2100F).

### Fabrication of glucose biosensor

For fabrication of the biosensor, at first 2 mg PANI–TiO_2_ composite was dispersed in 1 mL 0.5% chitosan solution prepared in 1% acetic acid and sonicated for 30 minutes. 1 mg of GOx was added to 250 μL of PBS and sonicated for well dispersion. After that 50 μL of the GOx solution was added to 50 μL of the previously prepared PANI–TiO_2_ dispersed solution and the mixture was sonicated for 30 min. Then, 5 μL of the resultant mixture was added on the dried surface of glassy carbon electrode (GCE) and after drying at room temperature, stored in refrigerator at 4 °C. 0.1% Nafion solution was used as binder for preparation of electrodes. All the electroanalytical experiments were carried out in a three-electrode system using N_2_ saturated PBS of pH 7.4 as electrolyte, Ag/AgCl electrode as reference electrode, surface modified GCE as working electrode and Pt foil as counter electrode. Cyclic voltammetry (CV) was carried out in a potential range from −0.7 V to +0.4 V taking 20 mL 0.1 M PBS solution in N_2_ atmosphere as electrolyte. For detection of glucose, a stock solution of 0.05 M glucose solution was prepared in a 0.1 M PBS solution of pH 7.4. Different volumes of the stock solution were added to the electrolyte to obtain the required concentration. The amperometric measurements were performed at a potential of −0.5 V.

### Experimental setup and photodegradation procedure

To carry out the photo-catalytic degradation study, we have used a photochemical reactor of jacketed quartz tube of length 21 cm. The inner diameter of the tube is 3.4 cm and the outer diameter is 4 cm. Mercury vapor lamp of 125 W purchased from Philips India has been used as the light source that predominantly radiated at 365 nm. After removing the outer shell of the bulb, it was placed carefully inside the quartz tube. To study the photocatalytic activity of the composite we have chosen rhodamine B (RB) as the model dye. As PANI-ES adsorbs anionic pollutants, we chose a cationic dye as a model pollutant to study the photocatalytic degradation. We have carried out the experiments using 25 mL of the dye solution using different initial concentrations (30 to 70 ppm) and photocatalyst dosages (15 to 25 mg). The solution was then stirred for 30 minutes in dark for adsorption and then irradiated with UV light for 2 h. Concentration of the RB solution was determined before and after the irradiation with UV light at different time interval by using UV/Visible spectrophotometer (UV-1800 Shimadzu model). It should be noted that each solution was 16 times diluted before recording the UV/Vis spectra.

## Conflicts of interest

The authors declare no competing interests.

## Supplementary Material

RA-010-D0RA01571G-s001

## References

[cit1] Lixia Y., Shenglian L., Qingyun C., Shouzhuo Y. (2010). A review on TiO_2_ nanotube arrays: fabrication, properties, and sensing applications. Chin. Sci. Bull..

[cit2] Nery E. W., Kundys M., Jelen P. S., Niedziolka M. J. (2016). Electrochemical Glucose Sensing: Is There Still Room for Improvement?. Anal. Chem..

[cit3] Ahmad M., Pan C., Luo Z., Zhu J. (2010). A Single ZnO Nanofiber-Based Highly Sensitive Amperometric Glucose Biosensor. J. Phys. Chem. C.

[cit4] Islam M. A., Atia M. A., Macka M., Paull B., Mahbub P. (2019). Electrochemical Characterisation of Nanoparticulate Zirconium Dioxide-on-Gold Electrode for Electrochemical Detection in Flow-Based Analytical Systems. Electrochim. Acta.

[cit5] Yang D. H., Takahara N., Lee S. W., Kunitake T. (2008). Fabrication of glucose sensitive TiO_2_ ultrathin films by molecular imprinting and selective detection of monosaccharides. Sens. Actuators, B.

[cit6] Atchudan R., Muthuchamy N., Edison T. N. J. I., Perumal S., Vinodh R., Park K. H., Lee Y. R. (2019). An Ultrasensitive Photoelectrochemical Biosensor for Glucose Based on Bio-Derived Nitrogen-Doped Carbon Sheets Wrapped Titanium Dioxide Nanoparticles. Biosens. Bioelectron..

[cit7] Cosnier S., Senillou A., Grätzel M., Comte P., Vlachopoulos N., Jaffrezic Renault N., Martelet C. (1999). A Glucose Biosensor Based on Enzyme Entrapment within Polypyrrole Films Electrodeposited on Mesoporous Titanium Dioxide. J. Electroanal. Chem..

[cit8] Bai J., Zhou B. (2014). Titanium Dioxide Nanomaterials for Sensor Applications. Chem. Rev..

[cit9] Pang X., He D., Luo S., Cai Q. (2009). An amperometric glucose biosensor fabricated with Pt nanoparticle-decorated carbon nanotubes/TiO_2_ nanotube arrays composite. Sens. Actuators, B.

[cit10] Si P., Ding S., Yuan J., Lou X. W., Kim D. H. (2011). Hierarchically Structured One-Dimensional TiO_2_ for Protein Immobilization, Direct Electrochemistry, and Mediator-Free Glucose Sensing. ACS Nano.

[cit11] Wang Z., Liu S., Wu P., Cai C. (2009). Detection of Glucose Based on Direct Electron Transfer Reaction of Glucose Oxidase Immobilized on Highly Ordered Polyaniline Nanotubes. Anal. Chem..

[cit12] Lai J., Yi Y., Zhu P., Shen J., Wu K., Zhang L., Liu J. (2016). Polyaniline-Based Glucose Biosensor: A Review. J. Electroanal. Chem..

[cit13] Zhu J., Liu X., Wang X., Huo X., Yan R. (2015). Preparation of Polyaniline–TiO_2_ Nanotube Composite for the Development of Electrochemical Biosensors. Sens. Actuators, B.

[cit14] Lee K. P., Komathi S., Nam N. J., Gopalan A. I. (2010). Sulfonated Polyaniline Network Grafted Multi-Wall Carbon Nanotubes for Enzyme Immobilization, Direct Electrochemistry and Biosensing of Glucose. Microchem. J..

[cit15] Shrivastava S., Jadon N., Jain R. (2016). Next-generation Polymer Nanocomposite-Based Electrochemical Sensors and Biosensors: A Review. Trends Anal. Chem..

[cit16] Zhu J., Huo X., Liu X., Ju H. (2016). Gold Nanoparticles Deposited Polyaniline–TiO_2_ Nanotube for Surface Plasmon Resonance Enhanced Photoelectrochemical Biosensing. ACS Appl. Mater. Interfaces.

[cit17] Tang W., Li L., Zeng X. (2015). A Glucose Biosensor Based on the Synergistic Action of Nanometer-Sized TiO_2_ and Polyaniline. Talanta.

[cit18] Daghrir R., Drogui P., Robert D. (2013). Modified TiO_2_ For Environmental Photocatalytic Applications: A Review. Ind. Eng. Chem. Res..

[cit19] Frank S. N., Bard A. J. (1977). Heterogeneous Photocatalytic Oxidation of Cyanide and Sulfite in Aqueous Solutions at Semiconductor Powders. J. Phys. Chem..

[cit20] Pruden A. L., Ollis D. F. (1983). Photoassisted Heterogeneous Catalysis: The Degradation of Trichloroethylene in Water. J. Catal..

[cit21] Thiruvenkatachari R., Vigneswaran S., Moon S. (2008). A Review on UV/TiO_2_ Photocatalytic Oxidation Process. Korean J. Chem. Eng..

[cit22] Akpan U. G., Hameed B. H. (2009). Parameters Affecting the Photocatalytic Degradation of Dyes using TiO_2_-Based Photocatalysts: a review. J. Hazard. Mater..

[cit23] Wei J., Zhang Q., Liu Y., Xiong R., Pan C., Shi J. (2011). Synthesis and Photocatalytic Activity of Polyaniline-TiO_2_ Composites with Bionic Nanopapilla Structure. J. Nanopart. Res..

[cit24] Reddy K. R., Karthik K. V., Prasad S. B. B., Soni S. K., Jeong H. M., Raghu A. V. (2016). Enhanced Photocatalytic Activity of Nanostructured Titanium Dioxide/Polyaniline Hybrid Photocatalysts. Polyhedron.

[cit25] Gilja V., Novakovic K., Travas-Sejdic J., Hrnjak-Murgic Z., Kraljic Rokovic M., Zic M. (2017). Stability and Synergistic Effect of Polyaniline/TiO_2_ Photocatalysts in Degradation of Azo Dye in Wastewater. Nanomaterials.

[cit26] Cheng Y., An L., Zhao Z., Wang G. (2014). Preparation of Polyaniline/TiO_2_ Composite Nanotubes for Photodegradation of Azo Dyes. J. Wuhan Univ. Technol., Mater. Sci. Ed..

[cit27] Emran K. M., Ali S. M., Al-Oufi A. L. (2018). The Electrocatalytic Activity of Polyaniline/TiO_2_ Nanocomposite for Congo Red Degradation in Aqueous Solutions. Int. J. Electrochem. Sci..

[cit28] Srivastava M., Srivastava S. K., Niralac N. R., Prakash R. (2014). A Chitosan-Based Polyaniline–Au Nanocomposite Biosensor for Determination of Cholesterol. Anal. Methods.

[cit29] Li K., Wang H., Pan C., Wei J., Xiong R., Shi J. (2012). Enhanced Photoactivity of Fe + N Codoped Anatase-Rutile TiO_2_ Nanowire Film under Visible Light Irradiation. Int. J. Photoenergy.

[cit30] Guo Y., He D., Xia S., Xie X., Gao X., Zhang Q. (2012). Preparation of a Novel Nanocomposite of Polyaniline Core Decorated with Anatase-TiO_2_ Nanoparticles in Ionic Liquid/Water Microemulsion. J. Nanomater..

[cit31] Srinivasu P., Singh S. P., Islam A., Han L. (2011). Novel Approach for the Synthesis of Nanocrystalline Anatase Titania and Their Photovoltaic Application. Adv. OptoElectron..

[cit32] Xua Q., Gu S. X., Jin L., Zhou Y., Yang Z., Wang W., Hu X. (2014). Graphene/Polyaniline/Gold Nanoparticles Nanocomposite for the Direct Electron Transfer of Glucose Oxidase and Glucose Biosensing. Sens. Actuators, B.

[cit33] Bao S. J., Li C. M., Zang J. F., Cui X. Q., Qiao Y., Guo J. (2008). New Nanostructured TiO_2_ for Direct Electrochemistry and Glucose Sensor Applications. Adv. Funct. Mater..

[cit34] Xie Y., Zhao Y. (2013). Electrochemical Biosensing Based on Polypyrrole/Titania Nanotube Hybrid. Mater. Sci. Eng., C.

[cit35] Cui H.-F., Zhang K., Zhang Y.-F., Sun Y.-L., Wang J., Zhang W.-D., Luong J. H. T. (2013). Immobilization of Glucose Oxidase
into a Nanoporous TiO_2_ Film Layered on Metallophthalocyanine Modified Vertically-Aligned Carbon Nanotubes for Efficient Direct Electron Transfer. Biosens. Bioelectron..

[cit36] Mahanta D., Manna U., Madras G., Patil S. (2010). Multilayer Self-Assembly of TiO_2_ Nanoparticles and Polyaniline-Grafted-Chitosan Copolymer (Cpani) for Photocatalysis. ACS Appl. Mater. Interfaces.

[cit37] Ahmad R., Mondal P. K. (2012). Adsorption and Photodegradation of Methylene Blue by Using Pani/TiO_2_ Nanocomposite. J. Dispersion Sci. Technol..

[cit38] Zhang H., Zong R., Zhao J., Zhu Y. (2008). Dramatic Visible Photocatalytic Degradation Performances Due to Synergetic Effect of TiO_2_ with PANI. Environ. Sci. Technol..

