# Supplementary material for: Deposition of an ultra-thin polyaniline coating on a TiO2 surface by vapor phase polymerization for electrochemical glucose sensing and photocatalytic degradation
Source: RSC Adv. 2020 May 5;10(30):17387–95. doi: 10.1039/d0ra01571g (PMC9053401; doi:10.1039/d0ra01571g)
Supplement: RA-010-D0RA01571G-s001 [file RA-010-D0RA01571G-s001.pdf]

Electronic Supplementary Material (ESI) for RSC Advances.
This journal is © The Royal Society of Chemistry 2020

Supporting Information

Ultra-thin Deposition of Polyaniline on TiO_2 Surface by Vapor Phase Polymerization for Electrochemical Glucose Sensor and Photocatalytic Degradation

Sibani Majumdar, Debajyoti Mahanta*

Department of Chemistry, Gauhati University,

Assam, India-781014

* To whom correspondence should be addressed

Dr. Debajyoti Mahanta

Tel: +91-6000682511

Fax: +91-0361-2700311

Email: debam@gauhati.ac.in, debam2@gmail.com

SI-1 Synthesis of polyaniline

For the preparation of Polyaniline (PANI), 5g aniline was dissolved in 150 mL 1 M HCl and stirred in an ice bath for half an hour. 12.25g APS was dissolved in 50 mL 1 M HCl and kept in an ice bath for 15 min and after that the APS solution was taken in a burette and added to the aniline solution drop wise under constant stirring and the reaction mixture was kept stirring for 6 h. The reaction was carried out at a temperature of 0-5°C. After completion of the reaction, the product obtained was washed with distilled water for several times and dried in air and then kept in a desiccator.

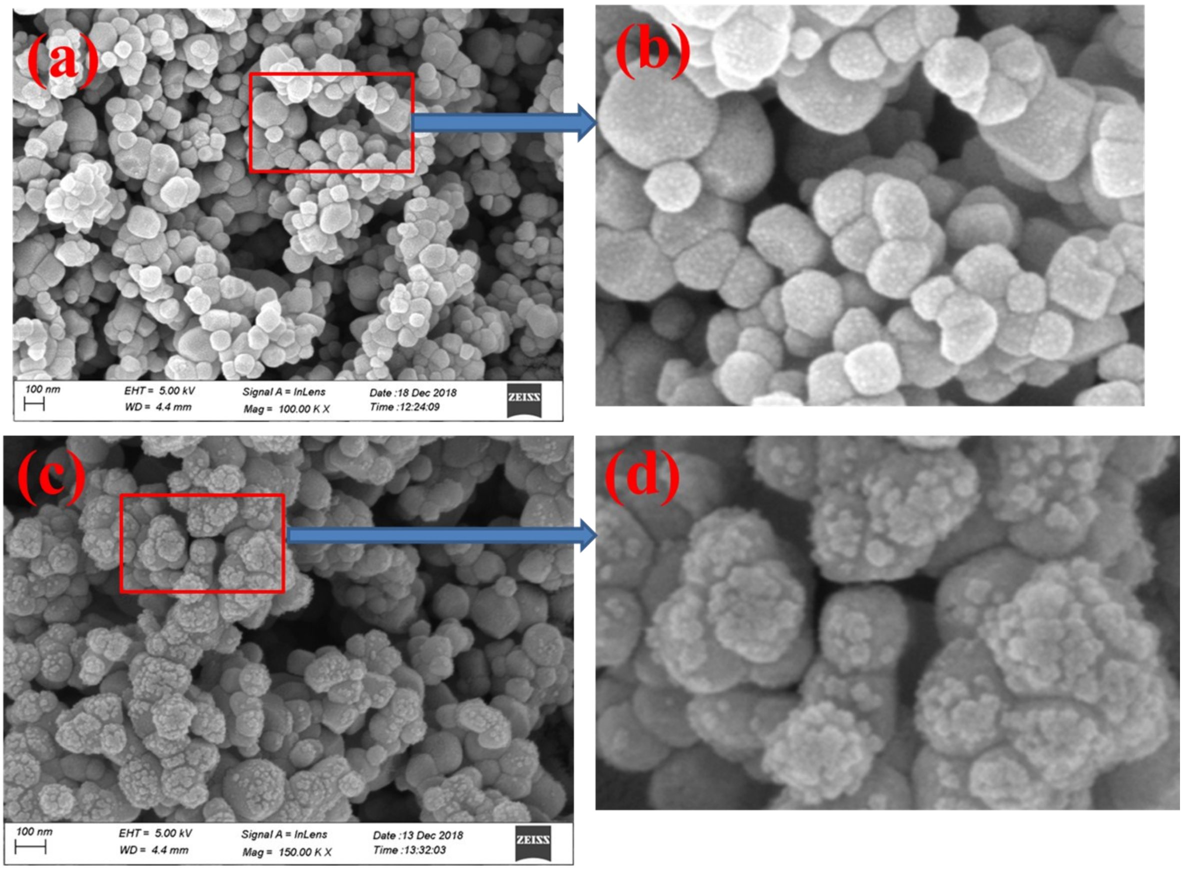

Figure S1: (a) SEM image of TiO_2 (b) zoomed image of TiO_2 particles (c) SEM image of PANI-TiO_2 (d) zoomed image of PANI-TiO_2
